# A Review on Environmental and Social Impacts of Thermal Gradient and Tidal Currents Energy Conversion and Application to the Case of Chiapas, Mexico

**DOI:** 10.3390/ijerph17217791

**Published:** 2020-10-24

**Authors:** Graciela Rivera, Angélica Felix, Edgar Mendoza

**Affiliations:** 1 Engineering Institute, National Autonomous University of Mexico, 04510 Mexico City, Mexico; griverac@iingen.unam.mx (G.R.); emendozab@iingen.unam.mx (E.M.); 2 National Council on Science and Technology, 03940 Mexico City, Mexico

**Keywords:** ocean renewable energy, OTEC, tidal current energy, environmental and social impacts

## Abstract

Despite the proved potential to harness ocean energy off the Mexican coast, one of the main aspects that have restrained the development of this industry is the lack of information regarding the environmental and social impacts of the devices and plants. Under this premise, a review of literature that could help identifying the potential repercussions of energy plants on those fields was performed. The available studies carried out around the world show a clear tendency to use indicators to assess impacts specifically related to the source of energy to be converted. The information gathered was used to address the foreseeable impacts on a hypothetical case regarding the deployment of an Ocean Thermal Energy Conversion (OTEC) plant off the Chiapas coast in Mexico. From the review it was found that for OTEC plants, the most important aspect to be considered is the discharge plume volume and its physicochemical composition, which can lead to the proliferation of harmful algal blooms. Regarding the case study, it is interesting to note that although the environmental impacts need to be mitigated and monitored, they can be somehow alleviated considering the potential social benefits of the energy industry.

## 1. Introduction

Governments worldwide are encouraging the development of projects for electricity production from renewable and clean sources to mitigate climate change, manage the possible reduction of fossil fuels, and ensure energy security [1]. The actual challenge is to reduce the gaps of knowledge in order to provide better and more precise information to the technology producers than the presently available. The main sources of ocean energy (OE) are tidal currents, tidal range, wave energy, OTEC (ocean thermal energy conversion), and salinity gradient [2]. In particular, the energy from tides and waves is arguably considered infinite [3] and they could constitute sufficient energy sources to supply the global demand. Therefore, governments and industry have expressed strong interest in them and by extension on all ocean energy sources. In 2008, Mexico approved its “Ley para el Aprovechamiento de Energías Renovables y el Financiamiento de la Transición Energética” (Renewable energy exploitation and energy transition funding law); as of its publication, the government decided to increase the allocation of public and private resources for research, development, and innovation in renewable energy (RE). This law was revoked in 2015, but it set the basis for planning and financing instruments for RE conversion technologies and aimed for the long term to cover the country’s urgent energy needs. RE-based generation is forecast to grow 6.8% annually with prospects of reaching 37.7% participation in the total Mexican generation by 2030 [4,5].

Today, the development of OE in Mexico is regulated and supported by the 2015 “Ley de Transición Energética” (LTE-Energy Transition Law). The scopes of the LTE are established in the “Ley General de Cambio Climático” (General law on climate change), where it is stated that, by 2020, the Ministry of Energy and the Energy Regulatory Commission should have an incentive system for electricity generation with RE promotion. In February 2020, the “Estrategia de Transición para Promover el Uso de Tecnologías y Combustibles más Limpios” (Strategy for a transition to promote the use of technologies and cleaner fuels) was published. There, the importance of creating economic incentives to encourage RE development is expressed again, but the mechanisms to do so are not specified [6]. The goals of maximum participation of fossil fuels expressed in the LTE are 65% in 2024, 60% in 2035, and 50% in 2050 [7,8]. Unfortunately, up to 2020 Mexico is still behind the commitments established.

Other Mexican laws involved in RE regulation are the “Ley Orgánica de la Administración Pública Federal” (Public federal administration law), the “Ley del Servicio Público de Energía Eléctrica” (Electricity public service law), and the “Ley de la Comisión Federal de Electricidad” (National electricity company law). This confirms the national commitment to incorporate RE technologies in the production of electricity, through social and environmental responsibility. The “Plan Nacional de Desarrollo” (National development plan), in the past 12 years, has considered sustainable development, including the promotion of the use of RE sources and technologies to face the challenges regarding diversification, energy security, and strengthen the development of science and technology [9].

In summary, Mexico has the natural power availability for energy generation from ocean RE and a robust regulatory framework to promote their development. However, an element that has delayed the installation of OE extraction devices is the uncertainty regarding the potential impacts of the energy plants on the environment and the cost of monitoring programs [10]. The studies available in the literature are still scarce and most of them evaluate only the impacts of prototypes with short operating periods, which has caused the construction permissions to be postponed or denied due to lack of information. This evidences that more efforts are needed to create legal and technological environmental frameworks. The present study shows an extended review of the literature related to possible environmental impacts of the implementation of plants to take advantage of the OE in Mexico. In particular, several coastal Mexican communities are not connected to the national electricity grid, which causes electricity to be of poor quality, extremely pollutant, unstable in availability, or even nonexistent. Two regions of the country where this problem is very clear are the Baja California peninsula (central part) and the Southeast Pacific (Guerrero, Oaxaca, and Chiapas). These three states present the greater social and economic development lag in the country [11]. The Ministry of Energy mentions that the state of Chiapas has more than 55,000 people without a connection to the electricity grid [12]. However, in this area of the country, wave energy is not considered sufficient for direct harvesting due to the low energetic waves found in the tropical region. For this reason, special emphasis has been given to tidal currents (TC) and thermal gradient (OTEC) sources around the southeast Pacific Ocean region of Mexico [13,14]. Moreover, these communities have the human right to adequate housing and improvement of their social welfare.

## 2. Methods

The ocean energy technologies are still at an early stage of development; therefore, uncertainties of the potential environmental impacts are one of the main obstacles for its deployment. Hence, through bibliographic analysis key factors of the marine environment were identified. Then, the methods that would allow quantifying or classification of the changes in the physical, chemical, or biological components on the water column were selected.

A wide overview of the potential impacts of OE technologies is provided. This means that our analysis seeks to link environmental factors to socioeconomic components, resulting in the environmental transformation by the establishment of energy infrastructure in the coastal zone. It should be noted that these areas are vulnerable as a result of the increase of anthropic pressure, which demands among other services, the supply of electricity for productive activities and living.

Finally, this information was used to carry out an analysis of the possible energy extraction on the coast Chiapas, to exemplify the elements that must be considered and how the unique characteristics of the sites can generate changes in the assessment of impacts, as well as focusing on areas with a high level in social marginalization.

## 3. Impact Assessment

### 3.1. Environmental Aspects

The review is based on environmental studies of OE devices, then the type of energy extraction was narrowed to OTEC (ocean thermal energy conversion) plants and tidal energy devices due to the physical and environmental conditions on the case study. The selection of the deployment site is crucial for project investors and it should be decided considering as many environmental factors as possible. In this section, the selected factors considered relevant are those that could trigger environmental perturbations in the area selected for the OE extraction site. Although any energy conversion plant deployment has some degree of impact, the identification of these elements is part of the strategic decision for project developers. This also helps in the process of public acceptance, political support, and reliability for future investments. The following selection aims to highlight crucial factors and methodologies to decrease uncertainty regarding OTEC and tidal energy projects [15,16,17,18,19,20,21,22,23,24,25,26,27,28,29,30,31,32]. This preselection will help identifying the potential impacts of the hypothetical case on the Chiapas coast.

The literature reviewed shows that the presence of dykes for the use of tidal energy and the establishment of tidal arrays, have the potential to induce significant changes in hydrodynamics. Factors such as reduction in the average flow, deviation of the tidal flow, increase in bottom drag, and variations in the height and phase of the tides have been reported [33,34]. Hence, when the flow velocity is reduced, the sedimentation of suspended particles increases [35].

Variation in the hydrodynamics has direct and indirect impacts on local populations of seabirds, fish, and mammals [21]. To prevent the decrease in kinetic energy, it is important to limit the amount of energy that is allowed to be extracted. According to existing reports, it is recommended not to exceed 10-20% of the net flow [35], whilst Betz’s limit of 60% should also be considered. Likewise, Kadiri et al. [33] documented that the acceleration of the flow in the vicinity of a turbine could lead to a resuspension of the sediments near the devices, which should be monitored to prevent modifications in the benthic communities.

The main impacts associated with OTEC technologies are deep water discharges, that can modify the trophic network. These can change the quantity and size of the species that are distributed in the areas surrounding the facilities of the plant [21]. Cilenti et al. [36] reported factors such as temperature, salinity, dissolved oxygen, chlorophyll, nutrients, and organic matter must be continuously monitored, to collect information in three periods: before establishment, during the construction, and the period of operation, to identify the potential modification of biota throughout the life cycle of the project.

In addition to abiotic factors, a local fauna inventory allows identifying changes in terrestrial and aquatic biota. Increases in the noise level produced during the construction and establishment of infrastructure can trigger the absence of key species, colonization, population decline, etc. An example of this, infrastructure suspended in the water column or on the seabed can attract organisms by changing the population structure, the colonization process occurs in the three phases of the life cycle of the project [25,37].

The increase in the level of noise produced by the construction or installation of the devices has repercussions on terrestrial and marine fauna. The monitoring of these changes is carried out by integrating several methods. Polagye et al. [35] proposed that the most feasible way to achieve monitoring at an acceptable cost is to incorporate various devices and technologies (e.g., video, sighting log, passive acoustic instruments). Davis [18] mentioned the use of sound velocity profiles collected by a CTD (conductivity, temperature, and depth sensor), where the degree of stratification of the water column can be observed and with physical–environmental modeling. In turn, Küsel et al. [38] estimated the propagation and the noise levels received in the environment). In other studies, hydrophones were placed over a long period, at a frequency of up to 50 kHz. This method was used on the central Oregon coast; the data obtained contributed to the elaboration of proposals to mitigate the possible impacts, such as population decline, alteration of migratory routes, acoustic disturbance, collision, among others [22].

Additionally, the presence of invasive species causes displacement or disappearance of local fauna, modifying the habitat permanently [37,39,40,41]. Therefore, it is crucial to obtain information about the species assemblages with a nonintrusive method. In several studies, remotely operated underwater vehicles (ROVs) are used to collect information on species composition, habitat associations, and population density [42,43,44], making it possible to identify colonization patterns in rigid structures, and use previous fauna records for comparison.

To define the monitoring method or devices it is necessary to know the foundation depth of the infrastructure [44]. For example, on the Big Russel Channel in the United Kingdom, the footage was used to compare species assemblages, thereby estimating abundance and dissimilarities between species at different sampling points [45]. Copping et al. [31] used the ecological risk model at the population level, incorporating the behavior and form of displacement to estimate fish mortality related to the establishment of turbines [25]. These models are complemented with in situ information on the distribution and behavior of the species.

Collision risk of marine mammals is a factor that slows the establishment of OE devices, this represents a crucial point in project decision-making. Cetaceans, being flagship species, are a group that increases the value of the system they live in, that is, they are species used as a symbol to attract public or government contributions to conservation programs [46]. Cetacean distribution patterns are estimated via echolocation with passive acoustic devices such as C-POD (Cetacean Acoustic Hydrophone Network). Thompson et al. [44] used this instrument within a range of 20-160 kHz for identifying dolphin distribution patterns. Despite technological support, both echolocation and group size estimates are complemented by visual identification [47].

The installation of OE devices arrays can be barriers limiting the movement of the species, leading in modifications of routes of migration. These potential variations are estimated using distribution, abundance, movement patterns, and satellite tracking data with 1-year periods under normal conditions [48]. MacKenzie [45] recommends that aerial, boat, and hydrophone monitoring should be performed for a minimum of 2 years after device placement. Finally, changes in the seabed due to drilling, excavation, installation of anchors, and power cables are also considered. Monitoring related to these activities should be carried out before and after construction to determine variations in the composition of the biota and should continue with a minimum period of 3 years [48]. It is crucial to collect information from the studies for the development of monitoring protocols [46], which contribute to the decision-making process.

### 3.2. Socioeconomic Aspects

According to Kerr et al. [47], to date, research on OE has focused on the evaluation of resources, device design, and environmental impact, thus concluding that social science research on these energies has had low priority. Changes in the landscape and the distancing of public opinion in infrastructure planning processes are factors that influence the acceptance of RE, this is linked to aspects of perception, economic impacts, or life quality [26,49].

The most commonly used components to assess socioeconomic impacts are cost–benefit analysis and environmental impact assessment. However, Uihlein and Magagna [2] mentioned that it is possible to quantify economic impacts at the regional level with empirical macroeconomic models, which allow quantifying the impacts on individual sectors, GDP (gross domestic product), the public budget, and household income. Kaldellis et al. [50] concluded that the social impacts in the pre-construction and post-construction of ocean wind energy and the coastal zone are as follows: the noise of the turbines, interruption of the landscape from the installation of the infrastructure, disturbance of the agricultural activities, and land-use change, as well as restriction of navigation routes that affect socioeconomic operations. With this, they demonstrated the importance of attaching the visual impact assessment and the marine landscape to the environmental impact assessment.

In an economic study carried out in England and Wales, Gibbson [51] revealed the reduction of the average house price between 5% and 6% in the presence of a visible wind farm at 2 km, falling to less than 2% between 2 and 4 km, in distances between 8 and 14 km, the reduction is close to zero. This trend could be reflected in the installation of energy extraction technologies in the coastal zones, whereas the modifications of the environment and the decrease in the beauty of the landscape, which is why it should be considered in socioeconomic studies.

An important social factor within technological developments is public participation during the planning and the decision-making processes since they can evoke opposition as a result of a lack of transparency through the project of OE [1]. Bonar et al. [32] suggest that the absence of public engagement and participation does not automatically lead to protests, however, the exclusion of the population in the decision-making process is inadequate since the inhabitants will be affected or benefited by these technologies.

## 4. Impacts of Specific Oceanic Energy Technologies

As noted, before, the impacts depend on the type of energy to be extracted, the shape, size, and number of devices as well as the anchors, etc. Mendoza et al. [52] offer an overview of the identification of impacts related to the biotic and abiotic variables of the environment with characteristics of the device to be installed. This section analyzes studies corresponding to the use of OTEC and TC [14,15,16,17,18,19,20,21,22,23].

### 4.1. Thermal Gradient (OTEC)

OTEC technology uses the temperature differences between the ocean surface water and cold water at a depth of 1000 m to activate a Rankine or similar cycle, and power is extracted from a gas driven turbine [53,54]. For this reason, high potential for OTEC projects exist in tropical zones. Recently, the research has focused on providing electricity and other goods to communities or islands with restricted access to national grids. OTEC plants can be closed-cycle (external working fluid), open-cycle (seawater as working fluid), and hybrid [16]. Additionally, platforms for OTEC plants can be onshore (land-based or near-shore) or offshore. There are significant cost variations in the plants due to the construction and maintenance needed by each. The floating platform installation has lower land use but requires submarine cables to carry the electricity to land, thus the cost of maintenance is higher comparatively with land-based plants [55]. The selection of the platform is crucial to determine the feasibility of the project. The plants need a pumping system as large quantities of deep cold water are carried through pipes to cool a working fluid, while surface water is used to heat it. After flowing through the system, the resulting discharge has different temperature and density than the surface water. The main impact of OTEC to the marine environment is related to the discharge plume (this volume can reach a few hundred cubic meters per second), its flow regime, and its trajectory. This plume can modify the availability of nutrients which, in turn, can favor eutrophication of the marine environment [36,56]. The difference in temperature and nutrient content could increase primary production in the surrounding environment, resulting in decreased dissolved oxygen levels and the possible proliferation of harmful algal blooms (HAB), so it is crucial to evaluate the depth of the plume discharge and the subsequent stabilization [57]. If the discharge is close to the surface, the difference in pressure will cause the release of gases, such as dissolved carbonates, which can lead to variations in the pH of the water column [48].

The increase of nutrient-rich deep ocean water on the surface may also be related to the proliferation of HAB. Giraud et al. [56] carried out evaluations of the effects of discharges from OTEC plants in the Martinique Islands; they found a variation of more than 0.3 °C at 150 m, concluding that the discharge would modify the phytoplankton assembly in the deep maximum of chlorophyll on a local scale. Additionally, the pumping system represents a threat to certain species, particularly those with little mobility that can be trapped and dragged [33,58].

A closed-cycle OTEC plant uses ammonia as working fluid due to its high thermal conductivity; tests have also been conducted with R404A, R717, R134A, among other fluids [58,59]. One concern related with the use of working fluids is the potential leakage as a result of seeping into the ocean if the pipes were damaged [58]; Golmen and Yu [60] point out that the risks related to ammonia will be low and manageable given the vast experience of handling this substance.

Garduño et al. [17] reported potential impacts related to the pipes of the OTEC plants, in depths between 100 and 150 m, such as industrial and sanitary discharges, the release of toxic coatings to the sea, drag, and compression of species.

### 4.2. Tidal Currents

The kinetic energy present in the currents can be harnessed using horizontal axis, transverse flow, or vertical axis turbines [2,61]. One of the main concerns for the placement of these devices is the local reduction of the tidal range since they can cause alterations in the vertical mixing of the water column and with it the increase of suspended particles, as well as the penetration of the light [62,63]. Increased light penetration and accretion pollutants from industrial, agriculture, or household discharges can alter water quality, which can lead to increased primary production and eutrophication [63].

The turbines used for energy extraction increase the underwater noise, causing stress and potential tissue damage to fish, marine mammals, and birds [64]. Frid et al. [61] mentioned that the effects can directly damage the sensory tissues or indirectly change the behavior of the individuals, some incidents of whale strandings have been associated to underwater noise from military activities. This information can link changes in the population dynamics of species sensitive to underwater noise and contribute to the proposal of prevention measures in the period of operation of OE devices [62]. Hammar [49] reports behavioral changes in species exposed to the noise produced by turbines at distances of 10 m, however, the evidence of this modification in behavior outside the laboratory is not conclusive. This exposes the need to carry out in situ studies during the operation of the impact determination device, to enable the definition of proposals for prevention and mitigation.

When working with tidal barrage, it is important to take into consideration whether or not there are migratory movements of organisms. Hooper and Austen [63] studied the effects on anadromous fish, due to the caused difficulty of arriving at the spawning area in freshwater, resulting in population decline. Other species with specific habitat requirements, vulnerable to changes in aquatic systems, can cause changes in the trophic network. Furthermore, the colonization of species in areas with anthropogenic disturbances must be taken into consideration and the establishment of invasive species [37]. According to Firth et al. [65] communities found in artificial structures are less diverse compared to natural habitats. Other impacts include risk of injury or collision of marine mammals and fish with the tidal barrages and turbines. To estimate the potential impact, it is common the use of encounter risk models (ERM), models of ecological risk at the population level, models of the time of population exposure (ETPM), collision risk models (CRM) and, encounter models, among others [19,20,32].

Finally, the literature mentions the use of submarine cables for the transport of energy, these emit electromagnetic fields (EMF), that could affect the behavior of marine biota. The impacts of EMFs depend on the magnitude and persistence of the field, while their effects could temporarily alter the direction of swimming or migration [66]. Gill et al. [67] mentioned that elasmobranchs, sea turtles, decapods, marine mammals, and teleost fish could present behavioral changes in the presence of emissions from submarine cables. Statistical evidence links stranding of marine mammals to high levels of EMF [66]. Therefore, laboratory studies are required to identify potential responses in organisms, the effect and impact thresholds of EMFs [30], as well as studies targeting key species.

Table 1 groups the methods for monitoring or evaluating changes in the environment as a result of the presence of OTEC and tidal current devices, as well as the minimum time recommended that monitoring must take to be reliable.

### 4.3. Strengths and Weaknesses of Published Literature

To corroborate de aforementioned crucial environmental factors in OTEC and tidal energy a search in SCOPUS database was made.

The displacement of water masses in the water column as a consequence of the discharges of OTEC plant is the main driver in potential changes in the marine environment. To understand and identify the variations in the OTEC plant surrounding oceanic models are used to simulate the trajectory and interactions of the OTEC discharge in the water column [17,69]. Thus, to date, there is a significant gap regarding the long-term modifications in the biological, physicochemical properties in the water column. Decision-makers play a major role in the development of the projects, in this process it is crucial to present net power generation, information on the potential environmental impacts, and cost-effective studies on the different OTEC platforms to make thermal energy competitive with other renewable energy sources.

**Figure 1:**
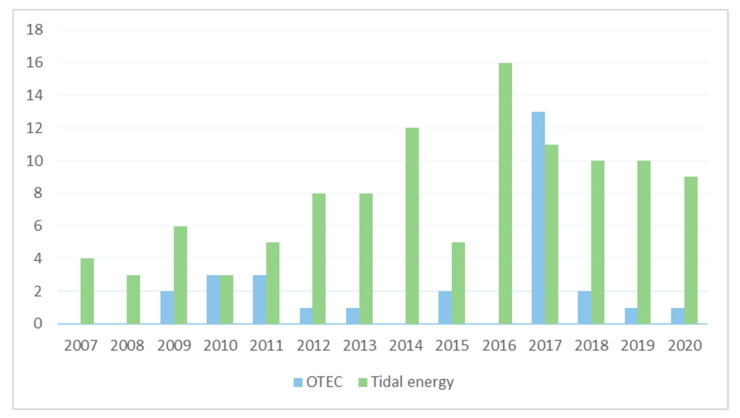
SCOPUS search of OTEC and tidal energy impacts in the environment, keywords; {OTEC} AND {environmental impact}, {tidal energy} AND {environmental impact}, {EMF} AND {marine energy}.

Figure 1 indicates the number of published studies related with OTEC, tidal and environmental impact assessment. This shows that OTEC is in an early stage of development, the main subject of the articles is simulations, investigation of alternative refrigerants, and recently the potential impact of this technology, however, is theoretical information. The lack of commercial development of OTEC plants is notable lack of commercial, or even large pilot plants to date certainly has relegated the study of large-scale implementation of this technology. Instead, available studies merely introduce comprehensive descriptions of the OTEC technology [17]. Commercial scale of the projects decreases uncertainties for the future investments and provides a general estimation of cost in the different stages of the projects (construction, operation and maintenance). Additionally, in this estimation the cost of the environmental assessment in all stages has to be added.

Tidal energy has a similar background, initially, design, estimation of energy output, optimizations of tidal turbines were the main subjects. The spatial overlapping between tidal turbines and marine mammals led to concerns with collision risk, interactions with infrastructure, and EMF potential changes in behavior are crucial for the future development of OE [70,71,72]. Potential negative effects have been considered in environmental assessment, at present, there is no empirical data on collision rates on operating turbines and the physical consequences, the data gathered is from numerical models that simulate coastal ocean processes, these simulations help to understand and identify potential changes in the marine food web [71,73,74]. Although modeling presents advantages to identify potential EMF effects, in-situ studies are scare making it difficult to determine alterations on the assemblage of species [53].

It is important to highlight that some studies made a quantitative evaluation (low, medium, and high), others focused on life cycle assessment and classified the potential effects in every stage of the projects [14,53,75].

In the last 3 years, the studies of OTEC projects have decreased while the number of published studies on tidal energy has remained constant. Nevertheless, in-situ information is scarce in both cases, thus delays the developments of the devices and reduces the opportunity to become cost-competitive in the market of renewable energy.

## 5. Case Study: OTEC Plant off the Coast of Chiapas, Mexico

Despite the government’s push in recent years to promote renewable energies, Mexico is advancing in small steps in the generation of renewable energies and more slowly in the extraction of OE. Progress is currently being made within the Mexican Center for Innovation in Ocean Energy (CEMIE-O), where in the last 3 years the work has focused on the definition of theoretical potentials for each type of energy that can be extracted [14], as well as, in detailed studies for those areas where a particular extraction seems possible. Examples of this are the use of ocean currents for the Cozumel channel [76]; studies of the thermal gradient for the eastern Pacific [77] or the waves in the northwest of the country [78].

**Figure 2:**
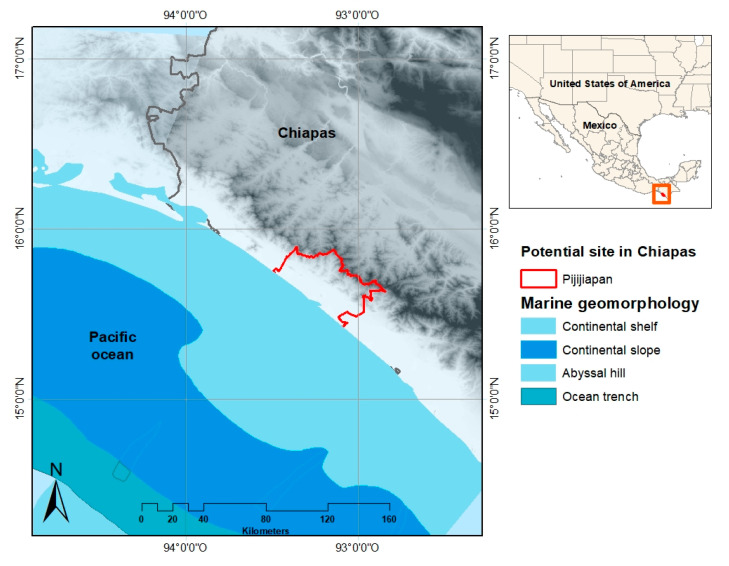
Potential site in the coast of Mexico for harnessing ocean thermal energy (OTEC)**.**

However, along the Mexican coast, it is possible to find small populations unconnected to the national electricity grid, this usually coincides with them being in a vulnerable condition to ocean threats. In the case of the coast of Chiapas as shown in Figure 2, 78% of the population is in poverty, this is broken down into extreme poverty with 29% and 49% with moderate poverty [79,80]. According to Borthwick [1,81] the main characteristic for an OTEC plant is to have a thermal gradient of 20 °C between the surface water and cold water from 1000 m depth. As reported by Garduño et al. [17] close to the coast of Chiapas the temperature difference is 21.45 °C; very close to the minimum usable gradient for OTEC.

The National Marine Renewable Energy Center of Hawaii estimated the potential on the Chiapas coast of 124.02 GW/h with a 100 MW OTEC plant and a sea surface temperature (SST) of 26.85 °C [81]. Yet, SAGARPA [82] reports an SST > 27 °C, measured from November 2017 to February 2018 obtained by satellite images of MODIS-AQUA of NOAA. The reported months are the coldest of the year, indicating the possibility of higher available power than the reported estimates of HINMREC. This corresponds with the results of autumn and winter thermal difference between 0 and 1000 m depth described by García et al. [77], in both seasons the differences were 25.17 and 23 °C, respectively, they used NOAA, NODC, ODV, and the Mexican Navy (SEMAR) database to analyze historical mean gradient in Mexico between surface and 1000 m depth.

Hernández-Fontes et al. [14] presented theoretical results of the availability of OTEC on the Mexican coast. In Chiapas, the authors estimated yearly percentages of >100, >150, and >200 MW of available power. Although electric power changes as a consequence of seasonal temperature variations, one of the best sites for the operation of the OTEC offshore plant is on the Chiapas coast [13]. However, the water pumping area is far from the coast and offshore plant studies are scarce to determine the feasibility.

The search for new energy sources is focused not only on being renewable, but also on being compatible with sustainable development. The government of Chiapas is committed to the conservation of its natural resources, so its development plan prioritizes environmental sustainability. In addition to this commitment, there is an interest in improving the quality of life of its population, and thus decrease its high level of marginalization.

### Potential Impacts of an OTEC Plant on the Chiapas Coast

From the analysis of the different studies cited in this article, the parameters that allow identifying changes in the physicochemical and biological structure of the water column were determined. These could lead to negative environmental impacts in the marine area, as well as in the coastal area during the three phases of the OTEC plant (construction, establishment, and operation), the impacts identified are summarized in Table 2.

Despite the lack of information on the effects of discharges in the surrounding areas of OTEC plants, there is knowledge of discharges of nutrients caused by other anthropogenic activities, for example, the NO_3_^−^ ion from residual discharges affects the aquatic invertebrates due to increased concentration and exposure time [83,84]. With this information, it is possible to compare the effects of the discharge plume of the OTEC plant on the surrounding environment.

Modifications of these factors would have a direct effect on the marine community [85,86,87,88]. Additionally, the Chiapas coast is a zone of upwelling, this has a relationship with the natural presence of HAB by the contribution of nutrients [85,86]. Notwithstanding, OTEC plume discharges could increase the frequency of these blooms, however, there are no studies that confirm this relationship.

It is crucial to monitor the changes associated with the HAB on the Chiapas coast since the increase of this community directly affects the fisheries, which are one of the main forms of income for the population. In addition, it also represents an alert for public health, even at a national level, different institutions in the health, production, and research sectors implement control methods for seafood contaminated with toxins to prevent the risk of poisoning [85,87].

As an offshore OTEC plant requires electric power transmission via marine cables, this can cause disturbances in the surrounding environment, as well as an increase in the capital cost for its establishment. The short-term environmental effects associated with cables include physical disturbances of the habitat as a result of their installation, resuspension of sediments. Together with the long-term effects (operational phase); heat emission, species colonization, and emission of electromagnetic fields [74].

The prediction of impacts by offshore plants can be supported by information on the construction of oil platforms, this may help with minimizing risks and better estimating costs. Nevertheless, the presence of the plant could cause social disagreement because of the visual impact on the landscape. This has been addressed by Gibbson [51] who mentions the decrease in property prices as a result of the presence of offshore wind farms.

Chiapas has mostly rural municipalities where more than 50% of the population lives in communities with less than 2500 inhabitants [88]. The high degree of social backwardness manifests itself in the coastal area, where irregular human settlements with less than 100 habitants predominate. Most of these groups are disconnected from the national electricity grid. Here, microgrids are an alternative energy supply for isolated communities, even the island mode would be appropriate [89]. This method of energy supply could strongly increase social welfare.

As a first step, an evaluation of consumption should be performed to identify the benefited communities [88]; in this case, the towns would be made up of El Fortín, Playa Cocos, and Las Conchas. These localities are located outside the polygon of the La Encrucijada biosphere reserve, therefore the installation of a microgrid does not pose a threat to the environment. The beneficiary population is of approximately 431 habitants. These localities are incorporated into the Program for the Development of Priority Zones, which seeks to provide basic housing services in localities with high levels of social backwardness in the country, however, information on consumption and electricity supply is incipient. Assuming that a 5–10 MW microgrid had the viable capacity to supply the aforementioned population, it is important to take into account that its control is decentralized and the maximum use of energy is limited [90].

With the supply of these three populations, it is possible to propose the extension for neighboring settlements, in such a way that it seeks to increase social welfare. In addition, this would allow areas for small-scale tourism; for example, Costa Azul Chocohuital previously presented problems due to the lack of infrastructure and services, minimizing the growth of the tourism sector. The expansion of energy supply would provide perks to both the tourism sector to increase hotel occupancy and basic housing services for the population. They are potential applications of the by-products of OTEC plants in the economic activities, for example, the use of deep water in aquaculture in this matter is necessary to select species adapted to low temperatures as salmonids their maturation is between 9 years at 13 °C and 13 years at 18 °C in the growth-fattening phases [91], also Masutani and Takahashi [92] mention the cultivation of oysters, lobster, abalone, kelp, and nori in aquaculture.

In the coast of Chiapas, 44.2% of the population carries out activities in the primary sector [93], mainly agriculture, livestock, and fishing. There are 606 artisanal fishermen distributed in four cooperatives, the main product is shrimp and scale [94]. For this reason, it is not possible to say with certainty that it is possible to integrate the by-products into the economic activities of the area, because fishermen are not familiar with the aquaculture of the aforementioned species. Furthermore, as a technology in development, studies on the use of these by-products are incipient, therefore, it cannot be affirmed that Mexican aquaculture would benefit from the supply of deep water.

Consequently, it is imperative to perform an analysis of the viability of using by-products from an OTEC plant, through the incorporation of courses, workshops, and capacitation to motive the involvement of the inhabitants.

The creation of jobs for the population close to the area where the energy plant will be displayed is one of the criteria to promote its placement. However, the characteristics of this job offer needs to include training given that the education level of the population aged 15 and over is of incomplete basic education (55.76%), and 13.47% are illiterate. In the stages of construction and establishment of an OTEC plant, it is possible to recruit workers from the population, however, in the operation phase, equipment and workers with specific knowledge of plant management and maintenance are necessary.

The direct impact on society is exclusively the supply of energy, and there would be no creation of any type of employment or the use of any added material. The direct impacts, related to visual obstruction, could be observed if they occur near towns with a high influx of tourism. Nonetheless, tourism is low compared to other areas of Oaxaca and the biggest limitation would be the rejection by the intrusion of external companies due to unacceptable practices of its predecessors.

**Figure 3:**
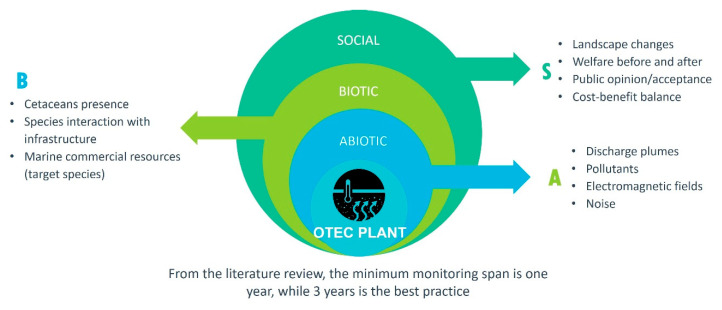
Aspects to be monitored for environmental and social impact assessment of ocean energies, OTEC Plant case.

Figure 3 shows a summary of the main aspects to be monitored before, during, and after the deployment of an OTEC plant in order to identify and assess the environmental and socioeconomic impacts it would produce off the Chiapas coast.

## 6. Conclusions

This study presented a first approach to the identification of basic factors (abiotic and biotic) that ocean energy projects associated with OTEC and TC should consider before, during, and after their deployment. The selection of these elements is a priority in the recognition of the problems that may arise in the specific area to be intervened and its current state. This information may contribute to the elaboration of monitoring protocols that allow the reduction or absence of negative impacts on the environment.

From the literature reviewed, two main conditions were found: (a) that an accurate assessment of environmental and socioeconomic impacts of ocean energy plants is not possible yet, due to the lack of installed facilities and (b) this does not mean that the development of the ocean energy industry should be left to a trial and error process. In a similar way to other coastal and marine projects, it is clear that monitoring is the path to understand the environment (its dynamics and resilience) and its response to the different drivers of change that the energy plants would produce along their useful life. Obviously, the main concern for investors is that systematic, permanent monitoring is an expensive activity so it must be planned and executed carefully. In this work an identification of the aspects to be monitored for tidal currents and OTEC plants was presented.

As shown in Table 1, the bibliographic compilation allowed defining the important variables to be monitored to detect changes in the biotic and abiotic factors associated with TC and OTEC. The modifications of these factors could lead to indicators for monitoring the response of the environment and can be used to support the impact assessment. The most important information presented, together with the variables, is the recommended method for data gathering and the minimum monitoring time recommended to produce time series long enough to detect changes and responses as well as undesired effects. Following these recommendations is seen as the path to effectively mitigate and correct any negative impact.

Notably, test sites play a key role in obtaining information from deployed devices, acting as centers for testing methods and data analysis focused on OE research programs. With this data, the effects can be extrapolated to different sites of interest and thereby promote the transition towards alternative energy.

The social and economic axis, as observed in the bibliographic search, is mainly related to cost–benefit studies, most published works do not consider the economic needs of the populations and their perspective in the potential changes in the surrounding environment. This review of information highlights the importance of conducting local studies of the benefits, social and economic, that the generation of OE will have on the population, to ingrate them in the decision process, and avoid possible future problems that put the project at risk. It is worth noting that monitoring is also applicable to socioeconomic variables. In this work, gathering of information regarding social welfare, services availability, public opinion on the energy plant and a general cost–benefit balance is proposed as the data framework for assessing the impacts of ocean energy on the local communities.

Despite having the optimal thermal gradient for the harnessing of thermal energy on the Chiapas coast, the extension of the continental shelf represents a limitation, coupled with the lack of studies of offshore plants that increase the uncertainty of their viability of maintenance costs. Environmentally, changes in the population structure of the water column and distribution of key species should be considered. Notwithstanding, this type of development must have a construction and production cost study to determine the economic viability of the establishment of the power plant.

## Figures and Tables

**Table 1:** Methods for monitoring potential impacts of Ocean Thermal Energy Conversion (OTEC) and tidal current (TC) into the marine environment, as well as the minimum monitoring period.

Factors		Technology		
**Abiotic**	Method	TC	OTEC	Period
**Hydrodynamic**	1D and 2D models, buoys, acoustic doppler velocimeter	x		1 year
**EMF**	Magnetometers, transects, gradiometer, calculation of the load Biot-Savart law	x	x	1–3 years
**Noise**	Hydrophones and sound velocity profiles	x	x	1 year
**Discharge plume**	Discharge plume model, ROMS (regional ocean models)		x	1 year
**Pollutant concentration**	Continuous stirred tank reactor	x	x	1 year
**Biotic**				
**Abundance of marine species**	Record of sightings, filming, remotely operated underwater vehicle, LIDAR, dives	x	x	3 years *
**Species interaction with infrastructure**	Geometric area model of risk rates and species interaction, predator–prey encounter model, multibeam sonar, exposure time, population model	x	x	1 year
**Cetaceans**	Echolocation C and T-POD, sightings records, radar	x	x	3 years
**Collision risk**	Acoustic and optical equipment complemented by sighting records	x		3 years **
**Collision risk seabirds**	Sightings records, visual recognition, radar, tracking devices	x		3 years **
**Collision risk fish**	Ecological risk model at population level, encounter risk model, radar	x		3 years **

* Macrofauna monitoring in certain seasons of the year, monitoring may be 1 year [68]. ** Related to the sighting of species, therefore the monitoring must be simultaneous.

**Table 2:** Methods to identify changes in the water column and the terrestrial part of the Chiapas coast.

Abiotic		Biotic	
Parameter	Method	Parameter	Method
Temperature	Multiparameter, NOAA data or use of MLD (mixed layer depth), CTD	Abundance of species	Diversity and species richness
Salinity	Multiparameter, MLD, refractometer, CTD	NOM-059-SEMARNAT-2010	Geographic Information System (GIS)
Dissolved oxygen	Multiparameter, Winkler’s method, CTD	Mangrove monitoring	Centered quadrant method
Nutrient	NO^2−^ Bendschneider method, NO^3−^ Stickland and Parsons method, NH^4+^ Koroleff method, orthophosphates method described by Murphy and Riley and total phosphorus Menzel and Corwin 13C/15N isotope technique	Chelonium distribution	Distribution data, quantification of nests and nesting females, and collection of morphological data
Chlorophyll	Spectrophotometry, satellite images	Vegetation analysis	1. NDVI (Normalized Vegetation Index) 2. SAVI (Soil Adjusted Vegetation Index)
Turbidity	Secchi disk or turbidimeter	Benthic fauna	Ekman dredge, nucleator, dives sampling
Suspended organic matter	Titration procedure	Primary production	Light/dark bottles, 14 C and satellite images

## References

[1] Borthwick G. (2016). Marine renewable energy seascape. Engineering.

[2] Uihlein A., Magagna D. (2016). Wave and tidal current energy—A review of the current state of research beyond technology. Renew. Sustain. Energy Rev..

[3] Siddiqui M.A., Ahmed S.M., Munir M.A., Hussain S.M., Randhawa J. (2015). Ocean Energy: The Future of Renewable Energy Generation. https://www.researchgate.net/publication/280937085_Ocean_Energy_The_Future_of_Renewable_Energy.

[4] INEEL (Instituto Nacional de Electricidad y Energias Limpias). https://www.ineel.mx/cemie-oceano.html.

[5] SENER (Secretaria de Energia). https://base.energia.gob.mx/Prospectivas18-32/PER_18_32_F.pdf.

[6] (2020). DOF (Diario Oficial de la Federacion). https://www.dof.gob.mx/nota_detalle.php?codigo=5585823&fecha=07/02/2020.

[7] DOF (Diario Oficial de la Federacion). http://www.diputados.gob.mx/LeyesBiblio/pdf/LGCC_130718.pdf.

[8] SENER (Secretaria de Energia). https://www.gob.mx/cms/uploads/attachment/file/62949/Prospectiva_del_Sector_El_ctrico_2013-2027.pdf.

[9] DOF (Diario Oficial de la Federacion). https://www.dof.gob.mx/nota_detalle.php?codigo=5565599&fecha=12/07/2019.

[10] Greaves D., Pérez C., Magagna D., Conley D., Bailey I., Simas T., Holmes B., O’Hagan A.M., O’Callaghan J., Torre-Encino Y. (2013). SOWFIA Enabling Wave Power: Streamlining Processes for Progress.

[11] CONEVAL (Consejo Nacional de Evaluacion de la Politica de Desarrollo Social). https://www.coneval.org.mx/Medicion/IRS/Paginas/Índice-de-Rezago-social-2010.aspx.

[12] SENER (Secretaria de Energia). https://datos.gob.mx/busca/dataset/regiones-sin-electricidad.

[13] García A., Rodríguez Y., Garduño P., Hernández E., Kim A.S., Kim H.J. (2020). General Criteria for Optimal Site Selection for the Installation of Ocean Thermal Energy Conversion (OTEC) Plants in the Mexican Pacific. Ocean Therm Energy Conversion—Past, Present Progress.

[14] Hernández-Fontes J.V., Felix A., Mendoza E., Rodríguez Y., Silva R. (2019). On the marine energy resources of Mexico. J. Mar. Sci. Eng..

[15] Scottish Natural Heritage (2016). Assessing Collision Risk between Underwater Turbines and Marine Wildlife. https://www.nature.scot/sites/default/files/2017-09/Guidance%20Note%20-%20Assessing%20collision%20risk%20between%20underwater%20turbines%20and%20marine%20wildlife.pdf.

[16] Collins N. (2012). Assessment of Potential Ecosystem Effects from Electromagnetic Fields (EMF) Associated with Subsea Power Cables and TISEC Devices in Minas Channel.

[17] Garduño E.P., García A., Rodríguez Y., Bárcenas J.F., Alatorre M.A., Cerezo E., Guadalupe J., Romero V.M., Silva R. (2017). Conversión de Energía Térmica Oceánica (OTEC), Estado del Arte.

[18] Davis A. (2012). Potential Impacts of Ocean Energy Development on Marine Mammals in Oregon.

[19] Kreting L., Elsaesser B., Kennedy R., Smyth D., O’Carroll J., Savidge G. (2016). Do changes in current flow as a result of arrays of tidal turbines have an effect on benthic communities?. PLoS ONE.

[20] Comfort C.M., Vega L. (2011). Environmental Assessment for Ocean Thermal Energy Conversion in Hawaii: Available Data and a Protocol for Baseline Monitoring.

[21] Knight C., McGarry S., Hayward J., Osman P., Behrens S. (2014). A review of ocean energy converters, with an Australian focus. AIMS Energy.

[22] Haverson D., Bacon J., Smith H.C.M., Venugopal V.X.Q. (2018). Modelling the hydrodynamic and morphological impacts of tidal stream development in Ramsey sound. Renew. Energy.

[23] Witt M.J., Sheehan E.V., Bearhop S., Broderick A.C., Conley D.C., Cotterell S.P., Crow E., Grecian W.J., Halsband C., Hodgson D.J. (2012). Assessing wave energy effects on biodiversity the Wave Hub experience. Philos. Trans. R. Soc..

[24] Wilde P., Sandusky J., Jassby A. (1978). Assessment and Control of OTEC Ecological Impacts.

[25] Dolman S.J., Green M., Simmonds M.P. (2006). Marine Renewable Energy and Cetaceans.

[26] Bender A., Francisco F.G.A., Sundberg J. A review of methods and models for environmental monitoring of marine renewable energy. Proceedings of the European Wave and Tidal Energy Conference.

[27] Jia Y., Nihous G.C., Rajagopalan K. (2018). An evaluation of the large-scale implementation of Ocean Thermal Energy Conversion (OTEC) Using an Ocean General Circulation model with low-complexity atmospheric feedback effects. J. Mar. Sci. Eng..

[28] Nihous G. (2018). A preliminary investigation of the effect of Ocean Thermal Energy Conversion (OTEC) effluent discharge options on global OTEC resources. J. Mar. Sci. Eng..

[29] Wood J., Joy R., Sparling C. (2016). Harbor Seal—Tidal Turbine Collision Risk Models: An Assessment of Sensitivities.

[30] Wilson B., Batty R.S., Daunt F., Carter C. (2007). Collision Risks between Marine Renewable Energy Devices and Mammals, Fish and Diving Birds.

[31] Copping A., Sather N., Hanna L., Whitting J., Zydleswki G., Stainesm G., Gill A., Hutchinson I., O’Hagan A., Simas T. (2018). Annex IV 2016 State of the Science Report: Environmental Effects of Marine Renewable Energy Development Around the World.

[32] Bonar P.A.J., Bryden I.G., Borthwick A.G.L. (2015). Social and ecological impacts of marine energy development. Renew. Sustain. Energy Rev..

[33] Kadiri M., Ahmadian R., Bockelmann-Evans B., Rauen W., Falconer R. (2012). A review of the potential water quality of tidal renewable energy systems. Renew. Sustain. Energy Rev..

[34] El-Gezinry T.M., Bryden I.G., Couch J.S. (2009). Environmental impact assessment for tidal energy schemes: An exemplar case study of the Strait of Messina. J. Mar. Eng. Technol..

[35] Polagye B., Copping A., Suryan R., Kramer S., Brown-Saracino J., Smith C. (2014). Instrumentation for Monitoring around Marine Renewable Energy Converters: Workshop Final Report.

[36] Cilenti L., Dario R., Dentamaro G., Di Lecce V., Guaragnella C., Cardellichio A., Mancinelli G., Petruzzelli D., Quarto A., Soldo D. Seawater distributed monitoring system: A proposal for architecture and data format. Proceedings of the IEEE International Conference on Environmental Engineering.

[37] Laidig T.E., Krigsman L.M., Yoklavich M.M. (2013). Reactions of fishes to two underwater survey tools, a manned submersibles and remotely operated vehicle. Fish Bull.

[38] Küsel E.T., Mellinger D.K., Thomas L., Marques T.A., Moretti D., Ward J. (2011). Cetacean population density estimation from fixed sensors using passive acoustics. J. Acoust. Soc. Am..

[39] Consoli P., Esposito V., Battaglia P., Altobelli C., Perzia P., Romeo T., Canese S., Andoloro F. (2016). Fish distribution and habitat complexity on Banks of the strait of Sicily (central Mediterranean Sea) from remotely operated vehicle (ROV) explorations. PLoS ONE.

[40] Culloch R., Bennet F., Bald J., Menchaca I., Jessop M., Simas T. (2015). Report on Potential Emerging Innovative Monitoring Approaches, Identifying Potential Reductions in Monitoring Costs and Evaluations of Existing Long-Term Datasets.

[41] Bald J., Curtin R., Díaz E., Fontán A., Franco J., Garmendia J.M., González M., Liriondo A., Liria P., Menchaca I. (2013). Guía Para la Elaboración de Estudios de Impacto Ambiental de Proyectos de Energías Renovables Marinas, Informe Técnico Realizado en el Marco del Proyecto Nacional.

[42] Sheehan E.V., Gall S.C., Cousens S.L., Atrill M. (2013). Epibenthic assessment of renewable tidal energy site. Sci. World J..

[43] Isasi-Catalán E. (2011). Los conceptos de especies indicadoras, paraguas, bandera y claves: Su uso y abuso en la ecología de la conservación. Interciencia.

[44] Thompson P.M., Brookes K.L., Cordes L. (2015). Integrating passive acoustic and visual data to model spatial patterns of occurrence in coastal dolphins. J. Mar. Sci..

[45] MacKenzie S. (2013). Techniques for Marine Biological Baseline Data Collection at Offshore Renewable Energy Developments and How Best Applty These to Guernsey Waters. Master’s Thesis.

[46] Shumchenia E.J., Smith S.L., McCann J., Carnevale M., Fugate G., Kenney R.D., King J.W., Paton P., Schwartz M., Spaulding M. (2012). An adaptive framework for selecting environmental monitoring protocols to support ocean renewable energy development. Sci. World J..

[47] Kerr S., Watts L., Colton J., Conway F., Hull A., Johnson K., Jude S., Kannen A., MacDougall S., McLachlan C. (2014). Establishing an agenda for social studies research in marine renewable energy. Energy Policy.

[48] Dreyer A., Polis H.J., Jenkins L.D. (2017). Changing tides: Acceptability, support, and perceptions of tidal energy in the United States. Energy Res. Soc. Sci..

[49] Hammar L., Sandén B. (2014). Will ocean energy harm marine ecosystem?. System Perspectives on Renewable Power.

[50] Kaldellis J.J., Apostolou D., Kapsali M., Kondoli E. (2016). Environmental and social footprint of offshore wind energy. Renew. Energy.

[51] Gibbson S. (2015). Gone with the wind: Valuing the visual impacts of wind turbines through houses prices. J. Environ. Econ. Manag..

[52] Mendoza E., Lithgow D., Flores P., Felix A., Simas T., Silva R. (2019). A framework to evaluate the environmental impact of ocean energy devices. Renew. Sustain. Energy Rev..

[53] Paredes M.G., Padilla-Rivera A., Güereca L.P. (2019). Life cycle assessment of ocean energy technologies: A systematic review. J. Mar. Sci. Eng..

[54] Soukissian T.H., Denaxa D., Karathanasi F., Prospathopoulos A., Sarantakos K., Iona A., Georgantas K., Mavrakos P. (2017). Marine Renewable Energy in the Mediterranean Sea: Status and perspectives. Energies.

[55] Cunningham J., Magdol Z., Kinner N. (2010). Ocean Thermal Energy Conversion: Assessing Potential Physical, Chemical and Biological Impacts and Risk.

[56] Giraud M., Garcon V., de la Broise D., L’Helguen S., Sudre J., Boye M. (2018). Potential effects of deep seawater discharge by an ocean thermal energy conversion plant on the marine microorganisms in oligotrophic waters. Sci. Total Environ..

[57] Khosravi A., Syri A., Assad M.E.H., Malekan M. (2019). Thermodynamic and economic analysis of a hybrid ocean thermal energy conversion/photovoltaic system with hydrogen-based energy storage system. Energy.

[58] Devault D.A., Péné-Annette D. (2017). Analysis of the environmental issues concerning the deployment of an OTEC power plant in Martinique. Environ. Sci. Pollut. Restor..

[59] Ganic E.N., Wu J. (1980). On the selection of working fluids for OTEC power plants. Energy Convers. Manag..

[60] Golmen L.G., Yu J.C.S., Dessne P., Golmen L. (2015). OTEC in the TROPOS multipurpose platform concept. OTEC Matters.

[61] Frid C., Andonegi E., Depestele J., Judd A., Rihan D., Rogers S.I., Kenchington E. (2012). The environmental interactions of tidal and wave energy generation devices. Environ. Impact Assess Rev..

[62] European Comission. http://publications.europa.eu/resource/cellar/3a4f6411-6777-11e7-b2f2-01aa75ed71a1.0001.01/DOC_1.

[63] Hooper T., Austen M. (2013). Tidal barrages in the UK: Ecological and social impacts, potential mitigation and tools to support barrage planning. Renew. Sustain. Energy Rev..

[64] Halvorsen M.B., Carlson T.J., Copping A.E. (2011). Effects of Tidal Turbine Noise on Fish Task 2.1.3.2: Effects on Aquatic Organisms: Acoustics/Noise—Fiscal Year 2011—Progress Report—Nvironmental Effects of Marine and Hydrokinetic Energy.

[65] Firth L.B., Thompson R.C., White F.J., Schofield M., Skov M.W., Hoggart S.P.G., Jackson J., Knights A.M., Hawkins S. (2013). The importance of water-retaining features for biodiversity on artificial intertidal coastal defence structures. Divers. Distrib..

[66] Fisher C., Slater M. (2010). Effects of Electromagnetic Fields on Marine Species: A Literature Review.

[67] Gill A.B., Gloyne-Philips I., Kimber J., Sigray P., Shields A.M., Payne A.I.L. (2014). Marine renewable energy, electromagnetic (EM) fields and EM-sensitive animals. Marine Renewable Energy Technology and Environmental Interactions.

[68] LaFrance M., English P., King J., Khan A. (2018). Benthic Monitoring during Wind Turbine Installation and Operation at the Block Island Wind Farm, Rhode Island.

[69] Finney K. (2008). Ocean thermal energy conversion. Guelph. Eng. J..

[70] Onoufriou J., Brownlow A., Moss S., Hastie G., Thompson D. (2019). Empirical determination of severe trauma in seals from collisions with tidal turbine blades. J. Appl. Ecol..

[71] Williamson B.J., Blondel P., Armstrong E., Bell P.S., Hall C., Waggitt J.J., Scott B.E. (2016). A Self-Contained Subsea Platform for Acoustic Monitoring of the Environment around Marine Renewable Energy Devices-Field Deployments at Wave and Tidal Energy Sites in Orkney, Scotland. J. Ocean Eng..

[72] Baker A.L., Craighead R.M., Jarvis E.J., Stenton H.C., Angeloudis A., Mackie L., Avdis A., Piggot M., Hill J. (2020). Modelling the impact of tidal range energy on species communities. Ocean Coast Manag..

[73] Scherelis C., Penesis I., Hemer M.A., Cossu R., Wright J.T., Guihen D. (2020). Investigating biophysical linkages at tidal energy candidate sites; A case study for combining environmental assessment and resource characterisation. Renew. Energy.

[74] Taormina B., Bald J., Want A., Thouzeau G., Lejart M., Desroy N., Carlier A. (2018). A review of potential impacts of submarine power cables on the marine environment: Knowledge gaps, recommendations and future directions. Renew. Sustain. Energy Rev..

[75] Zhang X., Zhang L., Yuan Y., Zhai Q. (2020). Life cycle assessment on wave and tidal energy systems: A review of current methodological practice. Int. J. Environ. Res. Public Health.

[76] Alcérreca-Huerta J.C., Encarnacion J.I., Ordoñez-Sánchez S., Callejas-Jiménez M., Barroso G.G.D., Allmark M. (2019). Energy yield assessment from ocean currents in the insular shelf of Cozumel Island. J. Mar. Sci. Eng..

[77] García A., Cueto Y., Silva R., Mendoza E., Vega L.A. (2018). Determination of the potential thermal gradient for the Mexican Pacific Ocean. J. Mar. Sci. Eng..

[78] Ocampo-Torres F.J. Wave Power Resources assessment in Northeast Mexico. Proceedings of the Pan American Marine Energy Conference.

[79] SEMARNAT (Secretaria de Medio Ambiente y Recursos Naturales) (2011). Política Nacional de Mares y Costas de México. Gestión Integral de las Regiones más Dinámicas del Territorio Nacional.

[80] CONAFOR (Comision Nacional Forestal) (2016). Programa de Inversión de la Región Istmo-Costa en el Estado de Chiapas.

[81] HINMREC (Hawaii National Renewable Energy Center). http://hinmrec.hnei.hawaii.edu/hinmrecftp/AnnualTempDiff.html.

[82] SAGARPA (Secretaria de Agricultura y Desarrollo Rural). https://www.gob.mx/cms/uploads/attachment/file/325216/Temperatura_superficial_marina_del_Pac_fico_Mexicano10nov17_02_feb_18.pdf.

[83] García-Mendoza E., Quijano-Scheggia S., Olivos-Ortiz A., Núñez-Vázquez E.J. (2016). Florecimientos Algales Nocivos en México.

[84] Camargo J.A., Alonso A. (2007). Contaminación por nitrógeno inorgánico en los ecosistemas acuáticos: Problemas medioambientales, criterios de calidad del agua, e implicaciones del cambio climático. Ecosistemas.

[85] Gárate-Lizárraga I., Pérez-Cruz B., Díaz-Ortíz J.A., López-Silva S., González-Armas R. (2015). Distribución del dinoflagelado Pyrodinium bahamense en la costa pacífica de México. Rev. Lat. Ambient Cienc..

[86] Ronsón J. (1999). Análisis retrospectivos y posibles causas de las mareas rojas tóxicas en el litoral del sureste mexicano (Guerrero, Oaxaca, Chiapas). Cienc Mar..

[87] Band-Schmidt C.J., Bustillos-Guzmán J.J., López-Cortés D.J., Núñez-Vázquez E., Hernández-Sandoval F. (2011). El estado actual del estudio de florecimientos algales nocivos en México. Hidrobiológica.

[88] Cota R., Velázquez N., González E., Aguilar A. Microrred aislada para una comunidad pesquera de Baja California, México: Caso de estudio. Proceedings of the IV Congreso Iberoamericano Sobre Microrredes con Generación Distribuida de Renovables.

[89] Marrero S. (2015). Estudio de Eficiencia Energética y Estabilidad de una Micro-red en La Restiga, isla de El Hierro. Master’s Thesis.

[90] Hossain E., Kabalci E., Bayindir R., Perez R. (2014). Microgrid testbeds around the word: State of art. Energy Conserv. Manag..

[91] Flores H., Vergara A. (2012). Efecto de reducir la frecuencia de la alimentación en la supervivencia, crecimiento, conversión y conducta alimenticia en juveniles de salmón del Atlántico Salmo salar (Linnaeus, 1758): Experiencia a nivel productivo. Lat. Am. J. Aquat. Res..

[92] Masutani S.M., Takahashi P.K., Steele J., Turekian K.K., Thorpe S.A. (2001). Ocean Thermal Energy Conversion (OTEC). Encyclopedia of Electrical and Electronics Engineering.

[93] ONU (2018). Índices Básicos de las Ciudades Prosperas, Medición, Nivel Básico.

[94] SAGARPA (Secretaria de Agricultura y Desarrollo Rural) (2010). Manifestación de Impacto Ambiental, Modalidad Particular para el Proyecto.

